# Epidemiological Mapping of Human Onchocerciasis in Transmission Suspected Districts of Bale, Borena, and West Arsi Zones of Eastern Ethiopia

**DOI:** 10.1155/2016/6937509

**Published:** 2016-08-25

**Authors:** Sindew Mekasha Feleke, Gemechu Tadesse, Kalkidan Mekete, Afework Hailemariam Tekle, Amha Kebede

**Affiliations:** ^1^Ethiopian Public Health Institute (EPHI), Addis Ababa, Ethiopia; ^2^African Program for Onchocerciasis Control (APOC), Ouagadougou, Burkina Faso

## Abstract

Onchocerciasis is mainly found in western part of Ethiopia and there is no evidence of transmission in the east ward. However, some zones (Bale, Borena, and West Arsi) are suspected for transmission given the area has fast flowing rivers and is covered with vegetation. Therefore, this study was conducted to map onchocerciasis transmission in those zones. About 19 villages were selected based on proximity to the rivers, representation of districts, zones, and vegetation covers, whereas the study participants, all village residents of age > 5 years with good health condition, were skin sniped and examined using microscopy. In this study a total of 2560 study participants were surveyed of which 1332 were female (52%) and 122 were male (48%). The age group of 21–30 years was highest (34.4%) and that of age > 51 years was the lowest (3.1%) study participants. The survey result revealed that none of the study participants regardless of age, sex, and location demonstrated skin snip* Onchocerca* microfilariae. The prevalence of microfilariae and community microfilarial load (CMFL) were 0% and 0 mf/s, respectively. The finding implied that there is no onchocerciasis in the area and, therefore, there is no need for interventions. Black fly distribution, cytotaxonomic study, and intraborder cross transmission monitoring are recommended.

## 1. Introduction

Onchocerciasis (river blindness) is a neglected tropical parasitic disease (NTD) caused by a filarial nematode worm called* Onchocerca volvulus* encapsulated in nodules under the skin. Female* Onchocerca volvulus* worms produce thousands of microfilariae which exit the nodules, move into the dermis, and enter the eye causing cutaneous and eye disease [1, 2]. Microfilaria picked up by black flies, also called* Simulium* flies, during a blood meal will develop into infectious stages (L3) and are transmitted to another person during subsequent bites [3, 4]. The disease is endemic in Latin America, Yemen, and Africa [5, 6]. Onchocerciasis causes high morbidity, psychosocial problems, and reduced agricultural productivity [3]. It is estimated that approximately 37.2 million people are infected and 1 million blinded or are visually impaired in 38 endemic countries around the world [3]. Ethiopia is one of the countries with high burden of onchocerciasis in the world. More than 28 million people who live in the surveyed endemic areas of Ethiopia are affected by the disease or are at high risk of infection [5]. However, there was no complete national mapping of onchocerciasis. Large parts of eastern Ethiopia remains unmapped although it is suspected for transmission; therefore, the full distribution of the disease and the status of transmission were not ascertained [5, 7]. The national onchocerciasis programme started in 2001 with the objective of controlling the disease to the level below public health importance. However, in 2012, the national onchocerciasis programme objective changed from control to elimination by interruption of transmission until 2020. The main strategy of elimination is by means of community based ivermectin (Mectizan®) distribution donated by Merck & Co. since 1987 to all who need it for as long as necessary [6, 8]. The annual or semiannual treatment of community using the drug ivermectin rapidly kills the microfilariae and reduces the fertility of adult female worms but does not kill them [1, 9–11]. Hence, it is used to control morbidity and to interrupt and ultimately eliminate transmission by clearing the skin microfilariae below the level needed for transmission by the black fly although there are flies biting. The goal of onchocerciasis elimination by 2020 necessitates the delineation of transmission boundaries of endemic areas and suspected adjacent districts. The disease is found, historically and as confirmed by several studies, in the northern, northwestern, western, and southwestern part of the country which have many flowing rivers and vegetation covers that favor fly breeding and are suitable for river blindness transmission. Although the ONCHOSIM prediction modeling showed that eastern Ethiopia is not suitable for onchocerciasis transmission [7] this study was conducted to confirm the transmission status with primary parasitological data collection. The national onchocerciasis elimination guideline also stated that the transmission status of onchocerciasis in the remaining part of the country needs to be checked if there is any low level transmission that potentially recurs later. The West Arsi, Bale, and Borena zones are administrative zones, previously unmapped, high vegetation coverage favorable for fly breeding, and were selected to undertake this survey.

## 2. Materials and Methods 

**Figure 1:**
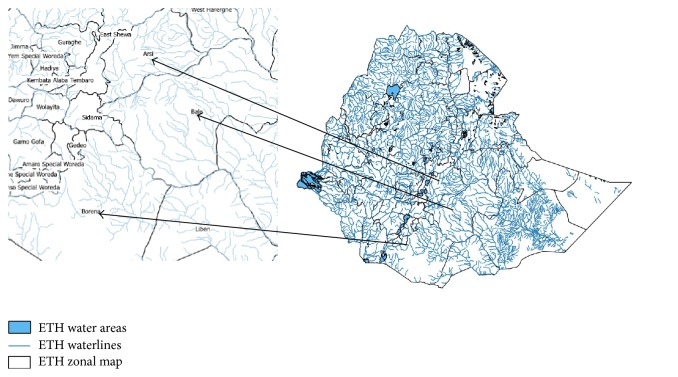
Zonal map of Ethiopia with water lines and study areas (Bale, Borena, and W. Arsi zones).

The survey was carried out in 19 villages found in 10 districts within 3 administrative zones of Oromia regional state (Bale, Borena, and West Arsi) (Figure 1) from September to October 2014. The area has mountains, flowing rivers, tributaries, and vegetation covers. The Bale mountain national park is also located in Bale zone. The villages were selected from each district based on close location along the river side as first-line community and representation of the districts. All permanent village residents above the age of 5 and with good health condition (not critically ill) were included in the study. The community sensitization and mobilization were done by the health extension workers and village leaders. The survey procedure was started first by holding meeting with the community and local authority members on the need and importance of the study, mobilizing them for full participation, and informing them about their right to decide to participate in the examination or not. Verbal informed consent was obtained at the point of registration and/or examination from all individuals, parents, or legal guardians before the commencement of the examination. After obtaining their consent, the registration of all household members of the village residents was carried out through interviews conducted with household heads of the selected villages or any of adult family members. The demographic information, clinical examinations (skin examination), and skin sample collection were carried out for each study participant. Using sterilized biopsy punch two skin snips were taken from left and right iliac crest from each household member above the age of 5. The skin sample was immediately transferred to the microtitration plate well containing normal saline solution and was kept at room temperature for 24 hours [2, 4, 12, 13]. The well numbers corresponding to the study participants were recorded on the patient information form. When all the 96 plate wells were full, the plate was sealed with a transparent adhesive tape and kept at room temperature. After 24 hours incubation, the fluid from each well was transferred to a slide and examined under a microscope for the presence of microfilaria under high power (40x) magnification [1, 10]. The results of the left and right iliac crest skin samples were recorded on the result form. All the demographic and parasitological data were entered and analyzed in Microsoft Excel and Epi Info, Version 3.1.1 CDC, USA.

The ethical approval for this study was obtained from Ethiopian Public Health Institute (EPHI) scientific and ethical review committee after the protocol being reviewed.

## 3. Results 

Out of the total 2560 study participants 1332 were female (52%) and 1228 were male (48%). The age groups from 21 to 30 years were the highest age group study participants (34.4%) and those of age > 51 years were lowest participants (3.1%) (Table 1). The results showed that no skin microfilariae of onchocerciasis were observed from any of 2560 study participants (Table 2). The prevalence of microfilariae and community microfilarial load (CMFL) by district, zone, and out of the total study participants were 0% and 0 mf/s in all 19 villages (Table 2). Skin rash and itching observed in eighty one (3.2%) study participants from Nensebo and Arena Buluq districts possibly associated with personnel hygiene and scabies.

## 4. Discussion

Several epidemiological studies of onchocerciasis were carried out by various investigators [14, 15] and established the prevalence from hypo- to hyperendemicity level in different parts of the country based on which elimination program implementation is instituted and ongoing [5, 13]. They have also reported the variation in prevalence with sex and age groups [16, 17]. The Ethiopian onchocerciasis elimination guideline [5] stated that an onchocerciasis implementation unit (districts) should be treated if the microfilarial prevalence is above 2%. In order to establish strong elimination programme, a complete map of the disease transmission boundary and community based ivermectin treatment with 100% geographical and >80% therapeutic coverage are basic steps to achieve elimination [2, 5, 14, 18]. In Ethiopia, large majority of the country's onchocerciasis transmission status was unknown [5, 7]. The national onchocerciasis elimination guideline [5] indicated the need to assess the transmission status in those unknown areas and identify intervention eligible communities and or document the findings for the country elimination dossier preparation if no transmission is ascertained in the area. However, our study result revealed that none of the skin samples demonstrated any* Onchocerca* microfilariae during microscopic examination with all age groups and sex of study subjects. Therefore, our study finding is important to inform policy makers that there is no onchocerciasis in the study areas; thus, intervention should be strengthened in known endemic adjacent transmission zones to enhance elimination and prevent expansion of transmission to nonendemic transmission free areas.

## 5. Conclusion

Based on the parasitological data, there is no human onchocerciasis in Bale, Borena, and West Arsi zones of eastern Ethiopia. Further operational research on onchocerciasis vector (*Simulium *fly) distribution assessment and xenomonitoring and cytotaxonomy study to monitor intraborder cross transmission is recommended.

## Figures and Tables

**Table 1:** Age category of study participants and microfilariae prevalence (Mf).

Age group (years)	<10	10–20	21–30	31–40	41–50	>50	Total
Number of study participants	120	502	881	710	268	79	2560
Age percentage	4.7	19.6	34.4	27.7	10.5	3.1	100
Mf prevalence	0	0	0	0	0	0	0

**Table 2:** Microfilaria (Mf) prevalence per village, districts, and zones.

Region	Zone	District name	Village name	Examined per village	Total positive	Prev. (%)	CMFL (%)	Lat	Lon
Oromia	*W/Arsi*	Dodolla	Keta	46	0	0	0	N0634.061	E03907.085
		Nensebo	Lemi	161	0	0	0	N0627.201	E03906.692
			Bochesa	114	0	0	0	N0525.771	E03814.642

Oromia	Borena	Dugdadawa	B/Megala	105	0	0	0	N0509.2241	E03718.783
		Teltele	Kelo	153	0	0	0	N0456.714	E03909.313
		Arero	Mekallu	80	0	0	0	N0456.715	E03852.738
			Oblo	117	0	0	0	N0353.628	E03813.325
		Dire	Corora	11	0	0	0	N0659.276	E03920.041

Oromia	Bale	Arena Buluq	Mida	206	0	0	0	N0625.750	E03934.672
			Oda Jila	185	0	0	0	N0623.90	E03937.15
			Magala	228	0	0	0	N62552	E0394049
		Dalo Mana	Wele	130	0	0	0	N0619.077	E03430.032
			M/Amana	137	0	0	0	N061457	E0394729
			Haye Oda	135	0	0	0	N62204	E0395443
		Mada Wollabo	Karu	126	0	0	0	NA	NA
			Arda Oda	206	0	0	0	N54939	E039329
		Berbere	Sirima	162	0	0	0	N64317	E401229
			Magala	148	0	0	0	N0647.319	E04007.338
			Umbella	110	0	0	0	N0636899	E04004129

*Total examined and the results*				*2560*	*0*	*0*	*0*		
